# Chronic Botulism in Humans: A Case Series

**DOI:** 10.1002/ccr3.71667

**Published:** 2025-12-14

**Authors:** Minoosh Shabani Barzegar, Sara Pourhemmati, Masoud Mardani

**Affiliations:** ^1^ Department of Infectious Disease, School of Medicine Loghman Hakim Hospital, Shahid Beheshti University of Medical Sciences Tehran Iran; ^2^ School of Medicine Shahid Beheshti University of Medical Sciences Tehran Iran

**Keywords:** botulism, case series, *Clostridium botulinum*, human botulism, neurologic manifestation, weight loss

## Abstract

This study presents novel clinical presentations of botulism, observed in both patients and goats from the same farm. Their symptoms appeared at the same time, suggesting a possible relation to animal visceral botulism. Interestingly, weight loss and weakness were the persistent signs, but not life‐threatening as expected in botulism.

## 1 Introduction

Botulism is a rare condition in humans as well as other mammals, including cattle, caused by neurotoxins produced by *Clostridium botulinum*. These toxins, called Botulinum neurotoxins known as BoNT, target neurons by cleaving the Soluble N‐ethylmaleimide Sensitive Factor Attachment Protein Receptor (SNARE) complex, resulting in inhibition of acetylcholine release in the neuromuscular junction [1, 2, 3]. There are seven types of BoNT. Types A, B, E, and, less frequently, F, are the dangerous types for humans, whereas type C is very frequent around the world among animals, which defines the rarity of human botulism [2, 4]. Chronic botulism refers to a proposed form of botulism where symptoms develop gradually and persist over time, rather than presenting acutely as in classic botulism. It has been described in animals, particularly cattle, under terms like “visceral botulism” [5]. In addition, animal‐to‐human transmissions are rare, but a few cases have been reported worldwide [4]. Different forms of the disease differ in the entrance root of the BoNT. The most common types include foodborne botulism, wound botulism, infantile botulism, and iatrogenic botulism. Adult intestinal botulism (so‐called botulism of undefined origin or visceral botulism in cattle) is like the infantile type caused by ingesting the spores, *C. botulinum* colonization, and releasing BoNT in mostly patients with gastric surgery, anatomical abnormalities, Crohn's disease, inflammatory bowel syndrome, long‐term antibiotic usage, and foodborne botulism because of bacterial imbalance in those patients [5, 6, 7]. The most frequently observed symptoms include xerostomia, diplopia, mydriasis, ptosis, facial paresis, reduced gag reflex, dysphagia, dysarthria, dysphonia, and cervical muscle weakness due to descending flaccid paralysis, which can progressively result in diaphragmatic paralysis and death. Also, there are many unusual presentations such as asymmetric muscle weakness, asymmetric ptosis, sensory deficits (rarely pain), non‐descending paralysis, gastrointestinal symptoms only, back pain, and difficulty using a walker, altered consciousness, and lip and tongue numbness [8, 9, 10].

## 2 Case History/Examination

Between March and May 2023, three members of a farmer family from Golestan province, Iran, presented with progressive neuromuscular complaints following consumption of homemade cheese produced from goats' milk with chronic botulism.

The first patient, a 35‐year‐old male farmer with no prior medical history, described 6 months of gradually worsening asthenia, beginning in the lower limbs and progressing to wheelchair dependence 3 days before admission. He reported 3 months of weight loss, persistent conjunctival redness with blurred vision, dysarthria, and xerostomia. He denied dysphagia, diplopia, gastrointestinal upset, sphincter dysfunction, or sensory disturbances. His symptoms began after regular consumption of the previously mentioned.

The second patient, his 59‐year‐old mother with hypertriglyceridemia controlled by 20 mg atorvastatin daily, presented initially on March 6th, 2023, with 4 months of dysphagia and odynophagia to both solids and liquids, progressive lower‐limb weakness, a tingling sensation in her hands and feet, and a 6–7 kg weight loss. She was discharged after supportive care when no diagnosis was confirmed, and botulism antitoxin testing was not performed at first admission. On April 15th, she re‐presented with worsening symptoms, and this time, further investigation was done.

The third patient, a 32‐year‐old brother, previously healthy, presented on May 14th, 2023, with 1 month of painful leg cramps and ocular discomfort but no visual restriction. His history was notable for the same dietary exposure, and he was aware of his relatives' prior admissions.

On physical examination, the first patient was wheelchair‐bound, while the second and third were ambulatory but weak. Bilateral conjunctivitis was present in the first and second patients, without ptosis, gaze palsy, or facial asymmetry. Pupils were mid‐sized and reactive in all three. The gag reflex was intact in each case, and no fasciculations were observed. Muscle tone was normal, but proximal weakness was evident: upper limbs 4/5 and lower limbs 3/5 in the first patient, symmetric 4/5 in the second, and 5/5 upper with 4/5 lower in the third. Deep tendon reflexes were depressed (1+) across all limbs.

## 3 Differential Diagnosis, Investigations and Treatment

Initial workups considered malignancy, rheumatologic disease, myasthenia gravis, Guillain‐Barré syndrome, and infectious causes such as HIV and brucellosis. The results of the workups are summarized in Table 1 and are available with full details in Appendix S1. Imaging, endoscopy, colonoscopy, and laboratory studies were unrevealing, despite aphthous lesions in the sigmoid and rectum of the first patient.

**TABLE 1:** Patients' laboratory test results.

Laboratory test/Paraclinical tool	Patient 1	Patient 2	Patient 3
White blood cells count (count in 10^3^ per μl^b^)	6.6	4.9	4.8
Hemoglobin (g/dl^c^)	13.3	11.9	13.6
Platelets (count in 10^3^ per μl)	200	189	194
Estimated sedimentation rate (mm/h^d^)	10	26	2
C‐reactive protein (mg/dl^a^)	12.6	2	1
7‐day blood culture/Double check	Negative/Negative	Negative/Negative	—
Albumin (g/dl)	4.9	4.9	4.1
Stool culture	No growth	No growth	—
Stool exam direct observation for parasites, fungus and ovals	Negative	Negative	Negative
Urine analysis for blood, hemoglobin, WBC, RBC and bacteria	Negative	Negative	—
Urine culture	No growth	No growth	—
Urine drug toxicology for amphetamines, morphine and cannabinoids	Negative	Negative	—
Coombs wright rapid test	Negative	Negative	—
Hepatitis B virus S antigen	Nonreactive	Nonreactive	—
Hepatitis C virus antibody	Nonreactive	Nonreactive	—
Human immunodeficiency virus antibody	Nonreactive	Nonreactive	—
Thyroid‐stimulating hormone (ng/ml^g^)	2.5	1.5	—
Peripheral blood smear	—	No significant pathology	—
Botulinum anti‐toxin in blood	Negative	Negative	Negative
Botulinum anti‐toxin in stool	Positive	Negative	Negative
Botulinum toxin in cheese sample	Positive	Positive	Positive
ENG‐MCV^e^	Normal	Normal	
Sonography	Fatty liver grade I	The uterus being retroflexed and atrophic, and a cystic region in the posterior wall of it with dimensions of 13 × 11 mm that OBGYN^f^ service found it not significant	
Endoscopy	Normal	Mild antral gastritis	
Colonoscopy	Normal	Internal hemorrhoid	
Chest CT‐scan^g^	Normal	A nodule with a diameter of 4 mm was observed in the left lower lobe of the lung, which the pulmonology service finds it not significant	
Brain CT‐scan		Nothing in favor of stroke	

^a^ Milligrams per deciliter.

^b^ Microliter.

^c^ Grams per deciliter.

^d^ Millimeters per hour.

^e^ Electromyography and nerve conduction velocity.

^f^ Obstetrics and Gynecology.

^g^ Nanograms per milliliter.

The second patient underwent a comprehensive evaluation during her first admission. At that time, no confirmed diagnosis was reached, and botulism antitoxin testing was not performed.

On April 15th, she re‐presented with aggravated symptoms. A more detailed history revealed the consumption of homemade cheese from goats with chronic illnesses, which were treated with a local herbal remedy named “seven leaves” for their muscle weakness and udder atrophy by a local vet. Botulinum toxin was detected in the cheese specimen. Stool specimens from the first patient tested positive for botulinum toxin at the Pasteur Institute, Tehran. Though BoTN was not detected in the stool or blood samples of the third and second patients, the shared exposure and clinical improvement strongly supported chronic botulism in all three.

Before antitoxin administration, each patient underwent an intradermal allergic reaction test, which indicated no hypersensitivity. According to national protocol, the initial adult therapeutic dose includes a minimum of 4500 IU for serotype A, 3300 IU for serotype C, and 3000 IU for serotype E. Given that the botulinum serotype was unknown in our patients, we used polyvalent antitoxin vials. Half of each dose was diluted in 100 mL of normal saline and administered via slow intravenous infusion over 30 min, while the remaining half was delivered intramuscularly.

The first patient received 2.5 vials on day one and 1.5 vials on day two, developing dysphagia that required ICU admission. The second patient, during her second admission, received the same regimen under ICU monitoring. The third patient received 2.5 vials on day one and 1.5 vials on day two, followed by an additional vial due to persistent symptoms.

## 4 Conclusion and Results (Outcome and Follow‐Up)

All three patients improved following antitoxin therapy, with resolution of asthenia, conjunctivitis, xerostomia, dysphagia, and weight loss. They were discharged in good and stable condition without adverse reactions. Diagnosis was confirmed by toxin detection in the first case and by clinical response in the latter two. Unfortunately, long‐term follow‐up could not be maintained due to distance and geographical limitations.

## 5 Discussion

Botulism is an uncommon but potentially lethal neuroparalytic disorder that requires rapid recognition and treatment. Although the classic presentation involves slurred speech, generalized weakness, bulbar paralysis, and dysphagia progressing to descending paralysis, a wide range of atypical features have been reported, including paresthesia, asymmetric paralysis, isolated gastrointestinal symptoms, back pain, altered consciousness, mental status changes, ascending paralysis, and lip or tongue numbness [9, 10, 11, 12, 13]. Chronic and recurrent forms have also been described. In 2007, recurrent botulism was reported in the United States after repeated ingestion of toxin from chili oil, with bulbar symptoms reappearing 6 months after initial therapy [14]. Similarly, prolonged toxin detection despite antitoxin administration has been documented in South Africa, where a patient required ventilation 14 weeks after diagnosis [15].

The acute form can progress rapidly and fatally. A Swiss case demonstrated severe cranial nerve impairment within 24 h [16], and patients with respiratory compromise were found to be 23 times more likely to die than those without [17]. For this reason, treatment must begin immediately upon suspicion, without waiting for confirmatory tests. Antitoxin therapy is most effective within 24–48 h of symptom onset [18], and intensive care support, including airway management, is essential.

Our cases add new observations to the clinical spectrum. Weight loss, not previously reported as a manifestation of botulism, was the primary complaint in one patient, alongside paresis. This initially suggested malignancy, stroke, diabetes, hypothyroidism, rheumatologic disease, or Parkinsonism, but all were excluded through imaging, laboratory testing, and endoscopic evaluation. Infectious causes such as brucellosis, HIV, hepatitis, and tuberculosis were also ruled out. Although BoNT was not detected in blood specimens, resolution of symptoms following antitoxin therapy strongly supported botulism as the underlying cause.

These findings suggest that extreme generalized weakness and chronic weight loss may represent under‐recognized manifestations of botulism. One possible mechanism is a systemic stress response to toxin exposure, leading to neuromuscular and metabolic dysfunction [19]. The prolonged recovery course and absence of alternative etiologies support this interpretation. Additionally, the absence of bulbar symptoms and the typical descending paralysis, as described in textbooks, were distinctive features of these cases.

Most food‐borne botulism cases arise from contaminated stored foods, but our series presented a different scenario. The patients were farmers consuming dairy from their own goats. Initial suspicion focused on cheese contamination during storage, supported by toxin detection in cheese samples. Interestingly, the goats themselves had shown chronic weakness and udder atrophy for months, treated locally with herbal remedies that improved weakness but not atrophy. This raised the possibility that contamination originated from the goats' milk rather than storage defects, or even direct animal‐to‐human transmission.

Although we lacked access to fresh milk for testing, literature confirms that BoNT can be detected in animal organs and, in rare cases, in milk [5]. Animal‐to‐human transmission is considered uncommon but has been reported worldwide [4]. Animals may also act as asymptomatic carriers, introducing toxin or vegetative forms into the food chain [20, 21]. Conversely, studies of farmers exposed to cattle with visceral botulism found no overlap in serotypes between humans and animals, suggesting transmission was unlikely, though cattle often harbor multiple serotypes that complicate interpretation [5]. A case–control study further questioned the concept of “chronic botulism” in cows, finding *C. botulinum* genes in both healthy and affected animals, with no clear link to human outbreaks [7].

In our series, animal‐to‐human transmission remains unconfirmed due to the absence of serotype comparison between goats and patients. Diagnosis relied on clinical suspicion and symptom resolution after antitoxin therapy. While intriguing, this hypothesis requires systematic investigation and controlled studies to clarify whether diseased animals can directly transmit botulism to humans.

The chronicity of symptoms in these cases was particularly notable, as the first case's symptoms began at least 5 months before proper treatment was received. This chronic course appeared rare given the typically rapid progression of botulism symptoms. In 2011, a paper on chronic botulism in farmers described human cases with symptoms such as weakness, fatigue, dry mouth, drowsiness, dizziness, nausea, and classic symptoms like blurred vision, difficulty swallowing, breathing difficulties, and speech problems. The article claims that chronic botulism can occur in humans via the same pathogenesis as infantile botulism. This means ingesting *C. botulinum* and subsequent colonization of the intestine can lead to in situ toxin production, resulting in chronic, non‐lethal symptoms of botulism in adults. This data may even explain previous cases reported as “botulism of undefined origin” worldwide [5]. However, the reports did not clarify why ingestion of *C. botulinum* is not problematic for all adults, prompting us to consider another finding in our second case. During malignancy investigations, colonoscopy in the second case revealed aphthous lesions which initially seemed unimportant. After reviewing the literature, this finding raises the important question of whether these lesions might facilitate *C. botulinum* colonization in the patient's colon, leading to chronic, non‐lethal symptoms through the sustained releases of small amounts of toxin over time. According to the literature, sub‐acute or chronic symptoms with less lethal outcomes are common in animals and may also occur in humans [5]. In animals, colonization of the intestinal tract by *C. botulinum* with continuous neurotoxin production leads to repeated exposure to small amounts of BoNT, which can provoke chronic botulism over time. The symptoms are often non‐specified, unlike the acute form of the disease, or may even be asymptomatic [12]. In humans, gradual toxin release due to *C. botulinum* intestinal colonization in adults or children over 1 year of age‐known as adult intestinal botulism‐has been rarely reported [6]. In animals, dysbiosis of normal intestinal flora and deficiencies in some trace elements appear to predispose to *C. botulinum* colonization [13]. Additionally, the presence of Enterococcus spp. has been shown to inhibit *C. botulinum* growth in the animal intestine [22]. Just as colonization can occur in animals due to dysbiosis, similar conditions‐such as dysbiosis or structural abnormalities like the aphthous lesions seen in our case‐may also enable colonization in humans. Accordingly, anatomical bowel abnormalities and Crohn's disease are mentioned in the literature as predisposing factors for adult intestinal botulism [6].

This report highlights three cases of botulism that were difficult to diagnose due to their chronic course and atypical presentation. Chronic weight loss and generalized muscle weakness, not previously described as manifestations of botulism, were observed in all three patients from the same farming family after consuming cheese produced from goats with suspected chronic botulism. Further evaluation suggested that intestinal anatomical abnormalities may have predisposed the patients to an uncommon form of adult intestinal botulism, characterized by a non‐lethal but prolonged course resulting from gradual exposure to small amounts of toxin. The potential link between chronic illness in animals and adult intestinal botulism in humans remains uncertain, and systematic studies are needed to clarify this relationship.

## Author Contributions

**Minoosh Shabani Barzegar:** conceptualization, methodology, project administration, writing – review and editing. **Sara Pourhemmati:** data curation, investigation, project administration, visualization, writing – original draft. **Masoud Mardani:** resources, validation.

## Funding

The authors have nothing to report.

## Consent

All three patients agreed to the application of their disease information anonymously for educational and research purposes, as written informed consent was obtained from the patients according to journal guidelines.

## Conflicts of Interest

The authors declare no conflicts of interest.

## Supporting information


**Appendix S1:** ccr371665‐sup‐0001‐AppendixS1.docx.

## Data Availability

The data that support the findings of this study are available on request from the corresponding author. The data are not publicly available due to privacy or ethical restrictions.
