# Supplementary material for: Chronic Botulism in Humans: A Case Series
Source: Clin Case Rep. 2025 Dec 14;13(12):e71667. doi: 10.1002/ccr3.71667 (PMC12703007; doi:10.1002/ccr3.71667)
Supplement: Supplementary file 1 — Appendix S1: ccr371665‐sup‐0001‐AppendixS1.docx. [file CCR3-13-e71667-s001.docx]

| LABORATORY TEST/ PARACLINICAL TOOL | PATIENT 1 | PATIENT 2 | PATIENT 3 |
| --- | --- | --- | --- |
| Urea (mg/dl^^[[1]](#footnote-1)^^) | 47 | 29 | 35 |
| Creatinine (mg/dl) | 0.8 | 0.6 | 1 |
| White blood cells count (count in 10^3 per µl^^[[2]](#footnote-2)^^) | 6.6 | 4.9 | 4.8 |
| Hemoglobin (g/dl^^[[3]](#footnote-3)^^) | 13.3 | 11.9 | 13.6 |
| Platelets (count in 10^3 per µl) | 200 | 189 | 194 |
| Sodium (mmol/l^[[4]](#footnote-4)^) | 137 | 137 | 136 |
| Potassium (mmol/l) | 3.8 | 4 | 3.8 |
| Iron (µg/dl^[[5]](#footnote-5)^) | 90 | 68 | - |
| Total iron binding capacity (µg/dl) | 286 | 269 | - |
| Ferritin (ng/ml^[[6]](#footnote-6)^) | 69 | 95 | - |
| Rheumatoid factor (IU/ml^^[[7]](#footnote-7)^^) | 17.1 | 19.1 | - |
| Estimated sedimentation rete (mm/h^^[[8]](#footnote-8)^^) | 10 | 26 | 2 |
| C reactive protein (mg/dl) | 12.6 | 2 | 1 |
| 7-day blood culture / double check | Negative / Negative | Negative / Negative | - |
| Alkaline phosphatase (IU/l^[[9]](#footnote-9)^) | 180 | 187 | 189 |
| Bilirubin total (mg/dl) | 0.5 | 0.5 | 1 |
| Bilirubin direct (mg/dl) | 0.1 | 0.1 | 0.3 |
| Creatinine phospho-kinase (IU/l) | 101 | 36 | 135 |
| Creatinine kinase-MB (IU/l) | 8 | 4 | 7 |
| Albumin (g/dl) | 4.9 | 4.9 | 4.1 |
| Troponin I qualitative | Negative | Negative | - |
| Stool culture | No growth | No growth | - |
| Stool exam direct observation for parasites, fungus and ovals | Negative | Negative | Negative |
| Urine analysis for blood, hemoglobin, wbc, rbc and bacteria | Negative | Negative | - |
| Urine culture | No growth | No growth | - |
| Aspartate amino-transferase (IU/l) | 20 | 18 | 20 |
| Alanine amino-transferase (IU/l) | 16 | 10 | 18 |
| Lactate dehydrogenase (IU/l) | 355 | 272 | 279 |
| Prothrombin time (seconds) | 12.9 | 10.7 | 12.8 |
| International normalized ratio | 1.12 | 0.84 | 1.11 |
| Partial thromboplastin time (seconds) | 28 | 25.6 | 26 |
| Urine drug toxicology for amphetamines, morphine and cannabinoids | Negative | Negative | - |
| Coombs Wright rapid test | Negative | Negative | - |
| 2 Mercoptoethanol titre | Negative (for the regional titre) | Negative (for the regional titre) | - |
| Indirect Coombs rapid test | Negative | - | - |
| Vitamin D (25 hydroxy calcitriol) (ng/ml) | 16 | - | - |
| Cytoplasmic anti-neutrophil cytoplasmic anti-body | Negative | Negative | - |
| Perinuclear anti-neutrophil cytoplasmic anti-body | Negative | Negative | - |
| Anti-β2-glycoprotein Immunoglobin G (IU/ml) | 0.2 | 0.1 | - |
| Anti-β2-glycoprotein Immunoglobin M (IU/ml) | 5.2 | 3.8 | - |
| Anti-Cardiolipin Immunoglobin G (IU/ml) | 0.7 | 0.1 | - |
| Anti-Cardiolipin Immunoglobin M (IU/ml) | 5.2 | 1.17 | - |
| Anti-topoisomerase I 1 (IU/ml) | 0.9 | 0.1 | - |
| Hepatitis B virus S antigen | Nonreactive | Nonreactive | - |
| Hepatitis C virus anti-body | Nonreactive | Nonreactive | - |
| Human immunodeficiency virus anti-body | Nonreactive | Nonreactive | - |
| Anti-cyclic citrullinated peptide (EU/ml^[[10]](#footnote-10)^) | 1.17 | 0.47 | - |
| Anti-nuclear anti-body | 0.7 | 0.3 | - |
| Anti-double stranded DNA (IU/ml) | 2.5 | 0.1 | - |
| Anti-Centromere - CREST (IU/ml) | 2 | 4.6 | - |
| Lupus Anti-Coagulant (IU/ml) | 41 | 35 | - |
| Thyroxine (ng/ml) | 7.7 | 8.3 | - |
| Triiodothyronine (ng/ml) | 1 | 0.9 | - |
| Thyroid stimulating hormone (ng/ml) | 2.5 | 1.5 | - |
| Parathyroid hormone (pg/ml^^[[11]](#footnote-11)^^) | 14.2 | - | - |
| Anti-Helicobacter Pylori Immunoglobin G (IU/ml) | 1.12 | - | - |
| Anti-Helicobacter Pylori Immunoglobin A (IU/ml) | 0.86 | - | - |
| Helicobacter Pylori stool Antigen | Negative | - | - |
| Peripheral blood smear | - | No significant pathology | - |
| Botulinum Anti Toxin in blood | Negative | Negative | Negative |
| Botulinum Anti Toxin in stool | Positive | Negative | Negative |
| Botulinum Toxin in cheese sample | Positive | Positive | Positive |
| ENG-MCV^^[[12]](#footnote-12)^^ | Normal | Normal | - |
| Sonography | Fatty changes of liver: Grade I | The uterus being retroflexed and atrophic, and a cystic region in the posterior wall of it with dimensions of 13*11 mm that OBGYN^^[[13]](#footnote-13)^^ service found it not significant | - |
| Endoscopy | Normal | Mild antral gastritis | - |
| Colonoscopy | Normal | Internal hemorrhoid | - |
| Chest CT-scan | Normal | A nodule with a diameter of 4 mm was observed in the left lower lobe of the lung that the pulmonology service finds it not significant | - |
| Brain CT-scan | - | Nothing in favor of stroke | - |

1. Milligrams per deciliter [↑](#footnote-ref-1)
2. Microliter [↑](#footnote-ref-2)
3. Grams per deciliter [↑](#footnote-ref-3)
4. Millimole per liter [↑](#footnote-ref-4)
5. Microgram per deciliter [↑](#footnote-ref-5)
6. Nanogram per milliliter [↑](#footnote-ref-6)
7. International Units per milliliter [↑](#footnote-ref-7)
8. Millimeters per hour [↑](#footnote-ref-8)
9. International Units per liter [↑](#footnote-ref-9)
10. Endotoxin Units per milliliter [↑](#footnote-ref-10)
11. Picograms per milliliter [↑](#footnote-ref-11)
12. Electromyography and Nerve Conduction Velocity [↑](#footnote-ref-12)
13. Obstetrics and Gynecology [↑](#footnote-ref-13)
